# Factors Related to Care Managers’ Experiences of Making Proxy Decisions about Older Adults Living Alone: A Cross-Sectional Study

**DOI:** 10.3390/nursrep13010006

**Published:** 2023-01-05

**Authors:** Hisao Nakai, Kuniko Ishii, Takako Sagino

**Affiliations:** 1 School of Nursing, Kanazawa Medical University, 1-1 Uchinada, Kahoku 920-0265, Ishikawa, Japan; 2 Faculty of Nursing, Hyogo University, Kakogawa 675-0195, Hyogo, Japan; 3 School of Nursing, Himeji University, Himeji 671-0101, Hyogo, Japan

**Keywords:** care managers, older adults living alone, decision-making

## Abstract

In Japan, the number of older adults living alone who require nursing care continues to rise. The purpose of this study was to identify factors associated with care managers’ experiences of making proxy decisions about life directions for older adults who live alone and whose intentions cannot be fully confirmed. The participants were care managers of in-home long-term care support providers nationwide. An original self-report questionnaire was created with reference to previous research and a web-based survey was conducted. The responses were obtained from 241 people and 211 people were included in the analysis. Two factors were identified that were related to care managers’ experiences of proxy decision-making about the life direction of older adults living alone whose intentions could not be fully confirmed: the ability to perform administrative tasks (odds ratio [OR] 3.38, 95% confidence interval [CI]: 1.39–8.22) and the observed cognitive deterioration (OR 2.89, 95% CI: 1.06–7.83). Even if older adults living alone can independently perform administrative tasks, observed cognitive deterioration may be a prodromal sign that such adults will be unable to make decisions about their future life.

## 1. Introduction

The global population is aging rapidly and the largest increase is in individuals aged over 65 years [1,2,3]. By 2025, the number of 65-year-old people in Asia may have doubled, with one in four people living in Europe and North America likely to be 65 or more years old [3]. In response to Japan’s aging population, a long-term care insurance system was launched in 2000, which covers nursing care services for older adults aged 65 years or over [4,5]. If an older person requires care services, they are assigned a care manager by the long-term care insurance system. The care manager creates a care plan for nursing care service use and supports the individual’s daily life [6]. Vulnerable homebound older adults rely on informal support as well as formal healthcare services [7]. Care manager follow-up is important, especially in the self-management of chronic diseases in older adults [8], as care managers are the most accessible and dependable care providers for older adults living alone. Many solitary older adults with chronic diseases rely solely on the visits and follow-up of their healthcare providers [7].

Older people with medical, cognitive, or social vulnerabilities are more vulnerable to rapid decline when faced with social isolation [9]. Additionally, the decision-making ability of older people is more complex. For example, in cancer treatment, the decision-making process is more complicated for older adults owing to physiological and functional changes caused by aging, social isolation, and functional impairment from complications and pre-existing diseases [10]. The International Consensus Group of the International Academy on Nutrition and Aging and the International Association of Gerontology and Geriatrics have recently provided definitions of cognitive frailty. Cognitive frailty has been defined as cognitive impairment owing to reduced cognitive reserve and is a clinical condition that coexists with physical frailty but is distinct from physiological brain aging [11]. The ability to differentiate between vulnerable and non-vulnerable older adults would enable physicians and healthcare providers to weigh the benefits and risks of interventions for older adults and could help patients to make better-informed choices [12]. Therefore, the observation and identification of cognitive frailty may be important in decision-making support for older adults.

For those older adults who live alone and do not have family to support them, the responsibility for support in end-of-life decision-making and advanced care planning falls on healthcare providers [13]. Care managers rely on their own experiences to judge the functioning of older adults living alone [14,15]. It has been pointed out that it is important for healthcare providers to anticipate future deterioration in cognitive function and confirm the intentions of older adults living alone with dementia [16]. Advance care planning interventions have a positive effect on the quality of end-of-life care for older adults [17,18] and can reduce stress, depression, and anxiety in surviving family members [19].

In Japan, more than 80% of care managers are involved in client decision-making on a daily basis [20]. The Japanese Ministry of Health, Labour and Welfare provides guidelines for decision-making support for older adults with dementia. These guidelines recommend that care providers build relationships with older adults with dementia from an early stage and emphasize the importance of care team interventions in the decision-making support process [21]. In contrast, there are no standard instructions or national guidelines for proxy decision-making. Care managers routinely request support from neighbors and families of older adults with cognitive disabilities [22]. Although care managers, other healthcare providers, and families are involved in decision-making support for older adults living alone, disagreements between these parties can cause further support to be more difficult [23]. Moreover, in Japan, neighboring residents sometimes notice changes in the daily routine of older adults living alone and report them to healthcare providers, who can then identify older adults who are experiencing difficulty living alone [24]. Care managers assess the cognition and functioning of older adults with cognitive impairment and older adults living alone by conducting regular visits [14,15], but there is no procedure for determining whether or not surrogate decision-making is necessary. Care managers are trusted to use their own experience to respond appropriately to issues that arise in the care of older adults.

It is difficult for older adults living alone to express their intentions in advance regarding the end of life. A study on decision-making support for terminally ill older adults living alone found that some care managers experienced distress when clients died without being able to confirm their intentions owing to disease progression [25]. It is quite difficult to encourage older adults living alone at home to anticipate their own cognitive decline unless there is a strong motivation to do so. It is important that care managers who support older adults living alone identify in a timely manner prodromal signs that clients may find it difficult to express their intentions in the future. The purpose of this study was to identify factors associated with care managers’ experiences of making proxy decisions about life direction for older adults who live alone and whose intentions cannot be fully confirmed. The findings may contribute to appropriate interventions for older adults living alone before they become unable to make decisions.

## 2. Materials and Methods

### 2.1. Terms Used in This Study

#### 2.1.1. Care Manager

In Japan’s long-term care insurance system, care managers are assigned to older adults who use long-term care insurance services. The care manager’s role is to create a care plan and support the needs of daily living for older adults [6,26].

#### 2.1.2. Data Collection

We conducted a web-based cross-sectional survey of 1002 care managers from in-home long-term care support providers nationwide. In-home long-term care support providers were selected by calculating the population ratio of each municipality using the 2020 population census and performing random sampling using Microsoft Excel. We emailed administrators of community-based services and asked them to circulate an invitation to participate in a survey of care managers. The questionnaire was an original anonymous self-administered questionnaire based on a survey report on care management by the Japanese Ministry of Health, Labour and Welfare. The aim of the questionnaire was to understand the practices of care managers and to use them to inform future policy [27,28]. The principal investigator developed draft survey items with reference to reports from the Ministry of Health, Labour and Welfare and all three researchers discussed and revised the draft questionnaire. We then asked three care managers from local governments who were involved in the research to test the draft questionnaire and provide their feedback. After two rounds of testing, three researchers discussed and revised the questionnaire. No statistical analysis was performed to test for reliability. The participants were informed about the purpose and significance of the research, the survey method, the fact that participation was voluntary, that participant responses were anonymous, and that individuals would not be identified even if they filled in the questionnaire. A letter of informed consent was distributed to the participants via email and the completion of the questionnaire implied their consent. The web-based survey was carried out using SurveyMonkey, a cloud-based survey development application. The care managers who agreed to participate accessed the survey using a QR code provided to them. The data were extracted in SPSS format from SurveyMonkey’s cloud system, which is ID and password protected. The survey was conducted from 11 July 2022 to 5 September 2022. The study is reported according to STROBE reporting guidelines for observational research [29,30].

### 2.2. Survey Contents

#### 2.2.1. Participant Background

Sex, age, years of experience, work type (full-time, full-time concurrent, part-time), and the total number of older adults living alone that participants were in charge of.

The participants’ experiences of the state of older adults living alone (multiple possible responses).

Difficulty communicating, inability to verbalize true intentions, careless behavior, observed cognitive deterioration, personality changes, refusal of care services, economic hardship, gambling interfering with life, and frequent complaints of anxiety. The response options were “no”, “a little”, “some”, and “yes”.

The participants’ experiences of life functions of older adults living alone (multiple possible responses).

Disposal of domestic waste, disposal of unwanted items, control of kitchen fires, food expiration date management, hospital preparation, pet care, planned shopping, and administrative tasks (e.g., city hall applications and banking procedures). The response options were “unable”, “somewhat unable”, “somewhat able”, and “able”.

#### 2.2.2. Socializing with Family and Neighbors

The tendency to prioritize the opinions of close relatives over the person himself/herself, complaints from neighbors, and troubles with neighbors. The response options were “no”, “not much”, “a little”, and “yes.”

The participants’ experiences of making a proxy decision on life direction while unable to fully confirm the individual’s intention.

The participants were asked, “Have you made proxy decisions about the direction of life for older adults living alone whose intentions you have not been able to fully confirm?”. The response options were “no” and “yes”.

### 2.3. Analytic Method

The data were analyzed for participants who responded to all subitems of the five categories described above (participant background, state of older adults living alone, life functions of older adults living alone, socializing with family and neighbors, and proxy decision-making experience).

To examine the factors related to care managers’ experiences of proxy decision-making about the life direction of older adults living alone whose intentions cannot be fully confirmed, we classified the data into two categories with reference to the Ministry of Health, Labour and Welfare’s 2018 survey on the revision of long-term care fees [31]. The care manager age was categorized as <50 years or ≥50 years. The years of experience was categorized as <10 years or ≥10 years. The work type was categorized as full-time or other (full-time concurrent, part-time). The number of older adults living alone that care managers were in charge of was divided into the categories <9 cases or ≥9 cases using the average value because this has not been investigated in previous studies. For care managers’ experiences of the state of older adults living alone, the responses for “none” and “very little” were combined into the category “none” and the responses for “some” and “yes” were combined into the category “yes”. For care managers’ experiences of the life functions of older adults living alone, the responses for “unable” and “somewhat unable” were combined into “unable” and the responses for “somewhat able” and “able” were combined into “able.” For relationships with family and neighbors, responses for “no” and “not much” were combined into “no” and responses for “a little” and “yes” were combined into “yes.”

To examine the relationship between an affirmative response to the statement “Have you made proxy decisions about the direction of life for older adults living alone whose intentions you have not been able to fully confirm?” and each item, the χ^2^ test or Fisher’s exact test was used.

A binomial multivariate logistic regression analysis was used to assess the factors associated with care managers’ experiences of making proxy decisions about the life direction of older adults living alone whose intentions could not be fully confirmed. Proxy decision-making experience was the dependent variable; sex, age, years of experience, and the number of older adults living alone that participants were in charge of were covariates. All the variables for which a statistically significant association (*p* < 0.05) was found in the univariate analysis were entered into the binomial multivariate logistic regression analysis using the forced input method. All the variables were entered after checking for multicollinearity (variance inflation factor ≥ 10). The significance level was set at 5%. IBM SPSS version 27 (IBM Corp., Armonk, NY, USA) was used for all the statistical analyses.

### 2.4. Ethical Considerations

This research was conducted in accordance with the Declaration of Helsinki, 1995 (as revised in Seoul, 2008) and was carried out with the consent of the university research ethics review committees at the authors’ universities (No. 22005). 

## 3. Results

The responses were obtained from 244 of the 1002 participants (response rate of 24.4%). Of the respondents, 211 had valid responses to all the items and were included in the analysis. There were 146 female care managers (69.2%) and 65 male care managers (30.8%). The average age (standard deviation) was 52.7 years (8.9), the average years of experience (standard deviation) was 11.6 years (5.7), and the average (standard deviation) number of cases of older adults living alone handled by care managers was 9.5 (7.4). Regarding the experience of making decisions on behalf of older adults living alone, 143 (67.8%) care managers said yes and 68 (32.2%) said no.

The results of the univariate analysis are shown in Table 1. The following variables were significantly associated with the experience of making a proxy decision on life direction for individuals whose intention could not be fully confirmed: Male sex (*n* = 51, 78.5%; *p* = 0.027), Careless behavior (*n* = 87, 75.7%; *p* = 0.007), Observed cognitive deterioration (*n* = 133, 71.9%; *p* = 0.001), Ability to dispose of domestic waste (*n* = 88, 73.9%; *p* = 0.029), Control of kitchen fires (*n* = 68, 75.6%; *p* = 0.037), Planned shopping (*n* = 65, 79.3%; *p* = 0.004), Administrative tasks (*n* = 130, 72.2%; *p* = 0.001), and Tendency to prioritize the opinions of close relatives over the person himself/herself (*n* = 128, 72.7; *p* = 0.001) (Table 1).

Table 2 shows the results of binomial multivariate logistic regression analysis with care managers’ experiences of making proxy decisions for older adults living alone as the dependent variable, after controlling for sex, age, years of experience, and number of older adults living alone that the participants were in charge of. The odds of the experience of making a proxy decision on the life direction for an individual whose intention could not be fully confirmed were 2.89 times greater when the client had cognitive deterioration versus when no cognitive deterioration had been observed (odds ratio [OR] 2.89, 95% confidence interval [CI]: 1.06–7.83). The care managers were 3.38 times more likely to make such a decision when the client could perform administrative tasks than when they could not (OR 3.38, 95% CI: 1.39–8.22) (Table 2).

## 4. Discussion

The purpose of this study was to identify the factors associated with care managers’ experiences of making proxy decisions about life direction for older adults who live alone. The care managers’ experiences of making such decisions were associated with older adults’ ability to perform administrative tasks and observed cognitive deterioration.

According to two 2020 reports, the average age and length of service of Japanese care managers are 45.1 years old and 9.7 years of service for men and 51.4 years old and 9.2 years of service for women [32,33]. The average age of the participants in this study was 52.7 years, the average years of experience was 11.6 years, and 67.8% had 10 or more years of experience. Although these data are similar to those in the two reports cited above, it is unclear whether our sample was representative of care managers in Japan.

Care managers need to be more proactive in identifying at-risk older adults for whom life plan proxy decisions are needed. A relative evaluation by care managers supporting older adults who live alone may identify cognitive deterioration, even in older adults able to perform administrative tasks such as city hall applications. In such cases, care managers may need to consider the possibility that the person will be unable to make future decisions about the direction of their own lives. The results of our univariate analysis suggest that even if care managers detect cognitive deterioration in older adults living alone, such adults may still be able to independently perform a range of tasks (e.g., household waste disposal, kitchen fire control, planned shopping). However, additional detailed studies are needed to explore the issues involved in such cases.

It is more likely that older adults with cognitive deterioration will require proxy decisions compared with older adults without cognitive deterioration. Therefore, care managers need to be able to detect cognitive decline earlier. A study in Japan by Shimada et al. showed that cognitive frailty may be a risk factor for dementia in older adults [34]. It is therefore desirable that care managers are familiar with the concept of cognitive frailty and that they can apply it to their observations of older adults. However, it is also important that older people living alone, even those with some cognitive deterioration, have a central role in life plan decision-making. Strategies are therefore needed to help care managers to support older people in making their own decisions.

During the support process, care managers may detect the signs of cognitive decline in older adults living alone before they experience difficulties in decision-making. If care managers can refer older adults living alone to medical institutions at an early stage, these clients may be able to begin timely treatment for cognitive decline. Early medical intervention may help to reverse cognitive decline in older adults. In the hospital treatment setting, if a patient lacks the ability to make decisions about treatment, an alternative decision-maker should be sought [35,36]. This is a more principled and appropriate attitude to decision-making support for older adults living alone. However, it is often the case that no proxy decision-makers are available for older people living alone who have lived in the community for many years. According to a survey by the Cabinet Office of Japan, approximately 18% of older adults living alone have no one to rely on if they become sick, approximately 13% do not want to rely on others, approximately 9% rely on healthcare providers, and 8% depend on friends or neighbors [37]. Overall, approximately 48% of older adults living alone have no one to seek help from if they become sick or plan to rely on someone other than relatives. Given the above, it is important for care managers to monitor older adults living alone for early signs of an inability to make decisions about life direction and adopt early preventative measures. The present findings may provide useful information about care managers’ decision-making support for older adults.

This study had some limitations. The collection rate for the survey, which targeted in-home nursing care support offices nationwide, was low at 24.4%, and the data were analyzed for only 21.1% of the initial sample. Although these results could inform attempts to anticipate decision-making difficulties in older adults living alone, the participants were care managers who were informed by their own practices and judgments. In other words, the necessity of making care decisions on behalf of older adults living alone was determined by the care managers; the intentions of the older adults they cared for were not examined. In addition, care managers were asked about cognitive deterioration in older adults living alone, but the effects of other mental and physical conditions and chronic diseases on cognition were not considered. This is an important issue that should be considered in future research. We did not control for the influence of any qualifications or clinical experience other than care manager qualifications. Therefore, these findings require validation by future studies on older adults living alone. The study used a cross-sectional design so causal relationships between the variables under investigation could not be established.

## 5. Conclusions

In decision-making support for the future life direction of older adults living alone, it is important to evaluate the ability to carry out everyday functions such as administrative tasks. However, of greater importance is the evaluation of cognitive function by a care manager who understands the previous cognitive function and characteristics of older adults. We recommend adding assessments of daily executive function and relative cognitive function to care manager monitoring.

## Figures and Tables

**Table 1:** Care managers’ experiences of making decisions on behalf of older adults living alone (*n* = 211).

				The Experience of Care Managers Making Proxy Decisions about the Direction of the Life of Older Adults Who Live Alone Whose Intentions Cannot Be Fully Confirmed				
Item	Category	Total		Yes		No		*p* Value
		N	%	N	%	N	%	
Care manager background								
Age (mean (standard deviation))	52.7 (8.9)	211	100	143	67.8	68	32.2	0.297
Years of experience (mean (standard deviation))	11.6 (5.7)	211	100	143	67.8	68	32.2	0.237
Number of older adults living alone in charge (mean (standard deviation))	9.5 (7.4)	211	100	143	67.8	68	32.2	0.739
Sex	Men	65	30.8	51	78.5	14	21.5	0.027
	Women	146	69.2	92	63.0	54	37.0	
Age	Less than 50	81	38.4	56	69.1	25	30.9	0.738
	50 years or older	130	61.6	87	66.9	43	33.1	
Years of experience	Less than 10 years	68	32.2	45	66.2	23	33.8	0.732
	10 years or more	143	67.8	98	68.5	45	31.5	
Number of older adults living alone in charge	Less than 9 cases	120	56.9	81	67.5	39	32.5	0.923
	9 cases or more	91	43.1	62	68.1	29	31.9	
Work style	Full-time	160	75.8	108	67.5	52	32.5	0.881
	Other (full-time, concurrent, part-time)	51	24.2	35	68.6	16	31.4	
The state of older adults living alone that the care manager has experienced.								
Difficulty to communicate	None	40	19.0	25	62.5	15	37.5	0.428
	Yes	171	81.0	118	69.0	53	31.0	
Cannot verbalize my true intentions	None	42	19.9	26	61.9	16	38.1	0.363
	Yes	169	80.1	117	69.2	52	30.8	
Careless behavior	None	96	45.5	56	58.3	40	41.7	0.007
	Yes	115	54.5	87	75.7	28	24.3	
Observed cognitive deterioration	None	26	12.3	10	38.5	16	61.5	0.001
	Yes	185	87.7	133	71.9	52	28.1	
Personality changes	None	101	47.9	66	65.3	35	34.7	0.470
	Yes	110	52.1	77	70.0	33	30.0	
Refusal of care services	None	68	32.2	40	58.8	28	41.2	0.055
	Yes	143	67.8	103	72.0	40	28.0	
Economic hardship	None	37	17.5	22	59.5	15	40.5	0.233
	Yes	174	82.5	121	69.5	53	30.5	
Gambling interfering with life	None	25	11.8	21	84.0	4	16.0	0.064
	Yes	186	88.2	122	65.6	64	34.4	
Frequent complaints of anxiety	None	172	81.5	117	68.0	55	32.0	0.870
	Yes	39	18.5	26	66.7	13	33.3	
Life functions of older adults living alone that care managers have experienced.								
Disposal of domestic waste	Cannot	92	43.6	55	59.8	37	40.2	0.029
	Able	119	56.4	88	73.9	31	26.1	
Disposal of unwanted items	Cannot	105	49.8	67	63.8	38	36.2	0.220
	Able	106	50.2	76	71.7	30	28.3	
Control of fire in the kitchen	Cannot	121	57.3	75	62.0	46	38.0	0.037
	Able	90	42.7	68	75.6	22	24.4	
Food expiration date management	Cannot	128	60.7	82	64.1	46	35.9	0.152
	Able	83	39.3	61	73.5	22	26.5	
Hospital preparation	Cannot	77	36.5	48	62.3	29	37.7	0.200
	Able	134	63.5	95	70.9	39	29.1	
Pet care	Cannot	191	90.5	127	66.5	64	33.5	0.219
	Able	20	9.5	16	80.0	4	20.0	
Planned shopping	Cannot	129	61.1	78	60.5	51	39.5	0.004
	Able	82	38.9	65	79.3	17	20.7	
Administrative tasks	Cannot	31	14.7	13	41.9	18	58.1	0.001
	Able	180	85.3	130	72.2	50	27.8	
Relationships with family and neighbors.								
Tendency to prioritize the opinions of close relatives over the person himself/herself	None	35	16.6	15	42.9	20	57.1	0.001
	Yes	176	83.4	128	72.7	48	27.3	
Complaints from neighbors	None	107	50.7	69	64.5	38	35.5	0.300
	Yes	104	49.3	74	71.2	30	28.8	
Trouble with neighbors	None	119	56.4	87	73.1	32	26.9	0.059
	Yes	92	43.6	56	60.9	36	39.1	
χ^2^ test								

**Table 2:** Factors related to care managers’ experiences of making proxy decisions about the life direction of older adults living alone whose intentions could not be fully confirmed.

			The Experience of Care Managers Making Proxy Decisions about the Direction of the Life of Older Adults Who Live Alone Whose Intentions Cannot Be Fully Confirmed		
Item	Category	OR	95% CI		*p*-Value
			Lower Limit	Upper Limit	
Sex	Man/Woman	1.96	0.91	4.22	0.085
Age	Less than 50/50 years or older	1.08	0.51	2.29	0.841
Years of experience	Less than 10 years/10 years or more	1.01	0.48	2.12	0.983
Number of older adults living alone in charge	Less than 9 cases/9 cases or more	1.55	0.79	3.05	0.206
Careless behavior	Yes/None	1.67	0.83	3.37	0.151
Observed cognitive deterioration	Yes/None	2.89	1.06	7.83	0.038
Disposal of domestic waste	Able/Cannot	1.09	0.52	2.28	0.811
Control of fire in the kitchen	Able/Cannot	1.08	0.51	2.31	0.836
Administrative tasks	Able/Cannot	3.38	1.39	8.22	0.007
Planned shopping	Able/Cannot	2.02	0.97	4.19	0.059
Tendency to prioritize the opinions of close relatives over the person himself/herself,	Able/Cannot	2.02	0.84	4.87	0.116

Binomial multivariate logistic regression analysis. OR: odds ratio; CI: confidence interval.

## Data Availability

The data presented in this study are available on request from the corresponding author.

## References

[1] World Health Organization Ageing and Health. https://www.who.int/news-room/fact-sheets/detail/ageing-and-health.

[2] United States Census Bureau An Aging World: 2015. https://www.census.gov/library/publications/2016/demo/P95-16-1.html.

[3] United Nations, Department of Economic and Social Affairs World Population Prospects 2019: Data Booklet. https://population.un.org/wpp/publications/files/wpp2019_databooklet.pdf.

[4] Ministry of Health, Labour and Welfare, Long-Term Care Insurance in Japan 2. The Long-Term Care Insurance System; 5. The Development of the Long-Term Care System Until Now and Future Issues. https://www.mhlw.go.jp/english/topics/elderly/care/index.html.

[5] Campbell J.C., Ikegami N., Kwon S. (2009). Policy Learning and Cross-National Diffusion in Social Long-Term Care Insurance: Germany, Japan, and the Republic of Korea. Int. Soc. Secur. Rev..

[6] Japan Care Manager Association What is Care Manager?. https://www.jcma.or.jp/?p=21659.

[7] Schenker M., Costa D.H. (2019). da Advances and Challenges of Health Care of the Elderly Population with Chronic Diseases in Primary Health Care. Ciênc. Saúde Coletiva.

[8] McCorkle R., Ercolano E., Lazenby M., Schulman-Green D., Schilling L.S., Lorig K., Wagner E.H. (2011). Self-Management: Enabling and Empowering Patients Living with Cancer as a Chronic Illness. CA Cancer J. Clin..

[9] Steinman M.A., Perry L., Perissinotto C.M. (2020). Meeting the Care Needs of Older Adults Isolated at Home during the COVID-19 Pandemic. JAMA Intern. Med..

[10] Soto-Perez-de-Celis E., Li D., Yuan Y., Lau Y.M., Hurria A. (2018). Functional versus Chronological Age: Geriatric Assessments to Guide Decision Making in Older Patients with Cancer. Lancet Oncol..

[11] Kelaiditi E., Cesari M., Canevelli M., Abellan van Kan G., Ousset P.-J., Gillette-Guyonnet S., Ritz P., Duveau F., Soto M.E., Provencher V. (2013). Cognitive Frailty: Rational and Definition from an (I.A.N.A./I.A.G.G.) International Consensus Group. J. Nutr. Health Aging.

[12] Clegg A., Young J., Iliffe S., Rikkert M.O., Rockwood K. (2013). Frailty in Elderly People. Lancet.

[13] Wakanako O., Tomoko N. (2021). Nationwide Survey Rerated to the Implementation Status of Bereavement Supports by Community General Support Centers in Japan: Characteristics of Community General Support Centers That Provided Bereavement Support. Jpn. J. Nurs. Sci..

[14] Hirakawa Y. (2017). Qualitative Study of Spirituality in End-of-Life Care for Elderly Men Living Alone: Perspective of Care Managers. J. Jpn. Assoc. Rural Med..

[15] Kubota M., Takayama S. (2017). Elderly with Dementia Living Alone -Personal Experiences Expressed by Elderly with Dementia and Accompanying Dangers Expressed by Care Support Specialists. Bull. Kansai Univ. Int. Stud..

[16] Hayashi J., Hayashi H., Zensho M., Zhang P. (2022). Support for Elderly People with Dementia Who Live Alone, According to the Stage of Progression of Dementia Symptoms, from Interviews with Nurses at a Community General Support Center. J. Jpn. Acad. Community Health Nurs..

[17] Sjöberg M., Edberg A.-K., Rasmussen B.H., Beck I. (2021). Documentation of Older People’s End-of-Life Care in the Context of Specialised Palliative Care: A Retrospective Review of Patient Records. BMC Palliat. Care.

[18] Brinkman-Stoppelenburg A., Rietjens J.A., van der Heide A. (2014). The Effects of Advance Care Planning on End-of-Life Care: A Systematic Review. Palliat. Med..

[19] Detering K.M., Hancock A.D., Reade M.C., Silvester W. (2010). The Impact of Advance Care Planning on End of Life Care in Elderly Patients: Randomised Controlled Trial. BMJ.

[20] Goto Y., Miura H., Ito N. (2022). Comparison between the Chief Care Manager and the Normal Care Manager on Hospitalization and Discharge Coordination Activities in Japan: An Online Cross-Sectional Study of Care Managers in Aichi Prefecture. Int. J. Environ. Res. Public Health.

[21] Ministry of Health, Labour and Welfare Decision-Making Support Guidelines in Daily and Social Life for People with Dementia. https://www.mhlw.go.jp/stf/seisakunitsuite/bunya/0000212395.html.

[22] Ando K., Mizuno K. (2015). Support from Care Managers to Elderly People with Moderate Level Dementia Living Alone Whose Family Lives in the Same Neighborhood. J. Jpn. Acad. Gerontol. Nurs..

[23] Sugihara Y., Yamada H., Komatsu M., Yamagata E., Okayama Y. (2016). Literature Review on Support by Care Managers in the Decision Making for Persons with Dementia. Doshisha Kango.

[24] Matsumoto K. (2021). Facilitation skills required of medical and welfare professionals in decision support for the elderly: Considering the process of supporting the elderly living alone in the use of care services. Study Soc. Relat..

[25] Oga Y., Mori A. (2018). Grief process associated with loss of role of care manager in home care support—From a survey on end-of-life support for elderly people living alone with cancer. Bull. Fac. Educ. Welf. Aichi Prefect. Univ..

[26] Tsutsui T., Muramatsu N. (2005). Care-Needs Certification in the Long-Term Care Insurance System of Japan. J. Am. Geriatr. Soc..

[27] Ministry of Health, Labour and Welfare Fact-Finding Report on the Development of a Multi-Layered Consultation Support System in the Community. https://www.mhlw.go.jp/content/12200000/000798785.pdf.

[28] Ministry of Health, Labour and Welfare The Investigative Research Report on How Care Managers Should Be in Order to Practice Better and More Effective Care Management, and on the Operation and Structure of Business Establishments That Provide Support. https://www.mhlw.go.jp/file/06-Seisakujouhou-12300000-Roukenkyoku/0000136653.pdf.

[29] STROBE. https://www.strobe-statement.org/.

[30] Cuschieri S. (2019). The STROBE Guidelines. Saudi J. Anaesth..

[31] Ministry of Health, Labour and Welfare The Investigation Related to the Verification of the Effect of the Revision of Long-Term Care Fees in 2018 and Survey Research. https://www.mhlw.go.jp/content/12601000/000500278.pdf.

[32] Statistics of Japan 2020 Wage Structure Basic Statistical Survey. https://www.e-stat.go.jp/stat-search/files?page=1&query=%E3%82%B1%E3%82%A2%E3%83%9E%E3%83%8D%E3%82%B8%E3%83%A3%E3%83%BC%E3%80%80%E5%B9%B3%E5%9D%87%E5%B9%B4%E9%BD%A2&layout=dataset&toukei=00450091&tstat=000001011429&metadata=1&data=1.

[33] Ministry of Health, Labor and Welfare Overview of the Results of the 2020 Wage Structure Basic Survey. https://www.mhlw.go.jp/toukei/itiran/roudou/chingin/kouzou/z2020/index.html.

[34] Shimada H., Doi T., Lee S., Makizako H., Chen L.-K., Arai H. (2018). Cognitive Frailty Predicts Incident Dementia among Community-Dwelling Older People. J. Clin. Med..

[35] Nakamura K., Kinugasa Y., Sugihara S., Hirai M., Yanagihara K., Haruki N., Matsubara K., Kato M., Yamamoto K. (2018). Sex Differences in Surrogate Decision-Maker Preferences for Life-Sustaining Treatments of Japanese Patients with Heart Failure. ESC Heart Fail..

[36] Torke A.M., Sachs G.A., Helft P.R., Montz K., Hui S.L., Slaven J.E., Callahan C.M. (2014). Scope and Outcomes of Surrogate Decision Making Among Hospitalized Older Adults. JAMA Intern. Med..

[37] Cabinet Office Fiscal 2014 Attitude Survey Results on Elderly People Living Alone (Overall Version). https://www8.cao.go.jp/kourei/ishiki/h26/kenkyu/zentai/pdf/s2-2.pdf.

